# 490. Uptake and Perceptions of COVID-19 Vaccines Among US Pregnant Women

**DOI:** 10.1093/ofid/ofab466.689

**Published:** 2021-12-04

**Authors:** Annette Regan, Pallavi Aytha Swathi, Marcianna Nosek, Ning Yan Gu

**Affiliations:** University of San Francisco, Orange, California

## Abstract

**Background:**

Compared to the non-pregnant population, pregnant persons are at increased risk for severe COVID-19 related illness, including higher rates of admission to intensive care and greater mortality. Despite the potential benefits of COVID-19 vaccines for pregnant persons, current guidelines for the use of COVID-19 vaccines during pregnancy are limited, and the uptake of COVID-19 vaccines among US pregnant adults is unclear.

**Methods:**

As part of an ongoing national longitudinal cohort study, 1,372 pregnant and recently postpartum pregnant persons participated in an online baseline survey, including questions on COVID-19 vaccination status and perceptions of COVID-19 vaccines. Preliminary analyses were restricted to 1,041 individuals who were pregnant during vaccine availability (after 14 December 2020). Post-stratification survey weights were applied to ensure results are representative of the general population. Weighted percentages and odds ratios were estimated based on survey responses.

**Results:**

39.4% (95% CI 33.7, 45.1%) of respondents received a COVID-19 vaccine during pregnancy. Predictors of vaccination included belief that COVID-19 was a serious disease (OR 2.49; 95% CI 1.41, 4.11) and concerns about giving birth during the COVID-19 pandemic (OR 1.83, 95% CI 1.10, 3.04). The most common reason for receiving a COVID-19 vaccine was to protect themselves (21.2%) or their baby (39.1%). Among unvaccinated respondents, 14.9% planned to receive a vaccine during their pregnancy and 35.3% after pregnancy, 28.6% had no intention of receiving a vaccine, and the remaining 21.1% were uncertain. Among those who never planned to vaccinate, the most common reason was concern about side effects (57.2%). Percent of pregnant persons receiving at least one dose of COVID-19 vaccine, by month of delivery (postpartum participants) or estimated month of delivery (pregnant participants).

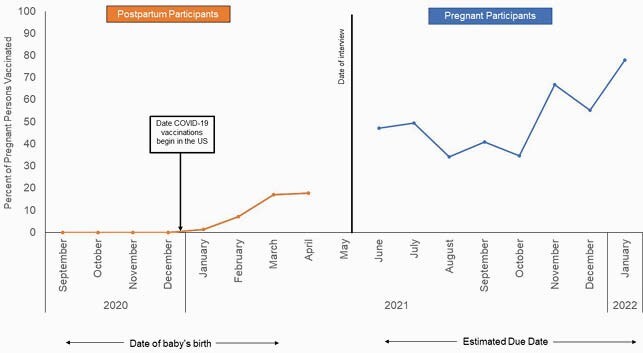

**Conclusion:**

Our results indicate that despite the lack of clear recommendations for vaccination during pregnancy, more than one-third of pregnant persons received a COVID-19 vaccine during pregnancy. Evaluation of the health effects of COVID-19 vaccination during pregnancy, including the ability to protect pregnant persons and their infants from infection, is needed.

**Disclosures:**

**All Authors**: No reported disclosures

